# Chinese Herbs Containing Aristolochic Acid Associated with Renal Failure and Urothelial Carcinoma: A Review from Epidemiologic Observations to Causal Inference

**DOI:** 10.1155/2014/569325

**Published:** 2014-08-27

**Authors:** Hsiao-Yu Yang, Pau-Chung Chen, Jung-Der Wang

**Affiliations:** ^1^Department of Occupational Medicine, Buddhist Tzu Chi General Hospital, No. 707, Section 3, Jhongyang Road, Hualien 97002, Taiwan; ^2^School of Medicine, Tzu Chi University, No. 701, Section 3, Jhongyang Road, Hualien 97004, Taiwan; ^3^Institute of Occupational Medicine and Industrial Hygiene, National Taiwan University College of Public Health, No. 17 Xuzhou Road, Taipei 100, Taiwan; ^4^Department of Environmental and Occupational Medicine, National Taiwan University Hospital and National Taiwan University College of Medicine, No. 1 Jen Ai Road, Section 1, Taipei 100, Taiwan; ^5^Department of Public Health, College of Medicine, National Cheng Kung University, No. 1, University Road, Tainan 701, Taiwan; ^6^Departments of Internal Medicine and Occupational and Environmental Medicine, National Cheng Kung University Hospital, No. 138, Sheng Li Road, Tainan 704, Taiwan

## Abstract

Herbal remedies containing aristolochic acid (AA) have been designated to be a strong carcinogen. This review summarizes major epidemiologic evidence to argue for the causal association between AA exposure and urothelial carcinoma as well as nephropathy. The exposure scenarios include the following: Belgian women taking slimming pills containing single material Guang Fang Ji, consumptions of mixtures of Chinese herbal products in the general population and patients with chronic renal failure in Taiwan, occupational exposure in Chinese herbalists, and food contamination in farming villages in valleys of the Danube River. Such an association is corroborated by detecting specific DNA adducts in the tumor tissue removed from affected patients. Preventive actions of banning such use and education to the healthcare professionals and public are necessary for the safety of herbal remedies.

## 1. Introduction

Epidemiology usually starts from careful clinical observation. In 1993, Vanherweghem et al. first reported an unusual observation that many young Belgian women took slimming pills containing Chinese herb subsequently developing renal failure and upper tract urothelial carcinoma [1, 2]. The slimming pills contained a Chinese herb “Guang Fang Ji” that has an off-label usage as a diuretic or an immune modulator [3]. At that time, the public generally believed that herbal products are harmless. However, based on the special pathological characteristics of extensive interstitial fibrosis of renal cortex without glomerular lesions, overwhelming upper tract urothelial carcinomas (UTUC), and identification of aristolochic acid (AA) in Chinese herbs [4], Vanherweghem and his colleagues hypothesized that ingestion of the* Aristolochia* species herbs may be the culprit for the epidemic in Belgium. Subsequently, Chinese herbs associating renal failure and urothelial cancer were reported in many countries [5–8]. Exposure to* Aristolochia* herbs has raised global public health concerns.

## 2. The Epidemiologic Approach

Because AA is derived from an extract of plants of the* Aristolochia*,* Bragantia*, and* Asarum* species, the further step is to determine whether an association between AA and the nephropathy exists in individuals who had similar exposures.

### 2.1. Exposures through Prescribed Chinese Herbal Products in Taiwan

AA is a common ingredient in many Chinese herbs, such as Ma Dou Ling (*Aristolochia debilis*), Tian Xian Teng (*Aristolochia contorta*), Qing Mu Xiang (*Aristolochia cucurbitifolia*), Guang Fang Ji (*Aristolochia fangchi*), Guan Mu Tong (*Aristolochia manshuriensis*), and Xixin (*Radix et Rhizoma Asari*) [9–11], and these herbal remedies are commonly prescribed for many different illnesses, including at least hepatitis, urinary tract infection, vaginitis, oral ulcer, upper respiratory tract infection, eczema, headache, dysmenorrhea, arthralgia, neuralgia, hypertension, cerebrovascular accident, bronchitis, pneumonia, heart failure, and edema [12]. In Taiwan, Chinese herbal remedies are regularly reimbursed through the national healthcare system, which covers more than 97% of the population [13]. By analyzing the National Health Insurance Reimbursement Database (NHIRD), Hsieh et al. found that herbal drugs containing AA had been prescribed to more than one-third of the population [14]. Besides, patients with a similar pathological finding of AAN in Belgium and history of taking Chinese herbal remedies containing AA were successively reported in Taiwan [5, 6, 15, 16]. As Taiwan was once reported to be the highest incidence of end-stage renal disease (ESRD) worldwide [17, 18], AA-associated nephropathy was therefore hypothesized to be one of the major risk factors for developing ESRD in Taiwan. Different from exposure to single Chinese herb in Belgium, Chinese medicine commonly applies mixtures of herbs to enhance efficacy and/or minimize toxicity [19, 20]. Lai et al. conducted a retrospective follow-up study using a systematic random sample from the NHIRD from 1997 to 2002 and found that the prescription of more than 30 g of Mu Tong or more than 60 g of Guang Fang Ji was associated with an increased risk of chronic kidney disease (CKD) in Taiwan [21]. This study explored the doses of prior exposure to AA containing Chinese herbal remedies and the occurrence of CKD and controlled confounding of use of nonsteroidal anti-inflammatory drugs and other risk factors to corroborate the causal hypothesis, indicating that mixtures of herbs did not reduce the nephrotoxicity of AA. In addition, Lai et al. also analyzed the NHIRD in Taiwan and found that people taking AA-containing herbal products had a dose-dependent increased risk of urinary tract cancer [22]. There is also an extremely high incidence of UTUC in Taiwan among hemodialysis patients [23], which was corroborated by cases from another hospital with added finding of high recurrence [24]. Although there was no statistical association between taking Chinese herbs and recurrence of cancer [24], it deserves further studies for effective prevention. Wang et al. followed the incidence of urothelial carcinoma among a national representative cohort of 58,739 patients with ESRD in Taiwan during 1997–2002 and reported significantly increased risks for UTUC (SIR = 11.6; 95% CI: 10.1–13.1) and bladder cancer (SIR = 13.9; 95% CI: 12.4–15.0) [25], which is later shown to be associated with the exposure to AA associated Chinese herbal products, especially Guan Mu Tong) [26]. Wang et al. further followed 90,477 newly diagnosed cases of ESRD in Taiwan between 1997 and 2008 covering the patients aged 40–85. Results showed that female patients had higher risk of UTUC than male after the diagnosis of ESRD; moreover, the cumulative incidence rates and the standardized incidence ratios of UTUC in females appear to decline after the calendar year of 2000 and the time trend is compatible with the decreased consumption of AA after 1998 [27]. In Taiwan, most AA-containing herbs have been prohibited since 2003. If AA is solely the causative agent for the epidemic in Taiwan, we expect that the incidence rate of UTUC in Taiwan will gradually decrease one decade after the cessation of exposure. Common Chinese herbs and formulas containing AA are summarized in Table 1.

### 2.2. Occupational Exposures of Chinese Herbalists in Taiwan

A different exposure setting is through the manufacturing of herbal products by Chinese herbalists. Chinese herbalist is a traditional occupation in Chinese society, and these individuals work in traditional herbal stores [28]. Yang et al. followed the mortality of urological cancers in 6,548 Chinese herbalists in Taiwan between 1985 and 2000, and the results showed that Chinese herbalists had significantly higher risks of mortality due to urological cancers (SMR = 3.10; 95% CI: 1.41–5.87) and due to chronic and unspecified nephritis, renal failure, and renal sclerosis (SMR = 2.40; 95% CI: 1.40–3.84) [29]. Yang et al. also followed the development of cancer until 2001; the SIRs for kidney and upper urinary tract cancers and bladder cancer were 4.24 (95% CI 2.47–6.80) and 2.86 (95% CI 1.52–4.89), respectively [30]. By assessing the herbalists' exposure at the individual level, Yang et al. found that manufacturing and selling Chinese herbal medicines as well as processing and dispensing herbal medicines containing AA increased the risk of renal failure and UTUC among herbalists due to the ingestion and inhalation of herbal powders at work [31, 32]. Because herbal particles are visible powders with large molecular weights, the exposure to AA was suspected to occur also via oral route, not only by ingesting herbal remedies containing AA but also by swallowing deposited powder particles in the oropharynx. This viewpoint seems to be supported by the highest risk of UTUC among workers who were exposed to grinding and packing procedures, which usually generates the most significant amount of airborne powders [32].

### 2.3. Exposure through Food Contamination in the Balkans

Balkan endemic nephropathy (BEN) is an environmental disease in farming villages in valleys of the Danube River. BEN and Chinese herbal AAN present similar clinical findings of nephropathy and urothelial carcinoma, which is characterized by slow, progressive renal failure with extensive hypocellular interstitial sclerosis, tubular atrophy, global sclerosis of the glomeruli, and cellular atypia and is also associated with UTUC [33]. These similarities with AAN led to the hypothesis that BEN was associated with dietary exposure to AA-contaminated bread when the seeds of* Aristolochia clematitis* are harvested along with the wheat used for bread making. In fact,* Aristolochia clematitis* is a plant that grows as a weed in endemic areas and contains AA [34], and the etiological evidence of epidemic was also corroborated by Grollman and Jelaković [35].

### 2.4. Evidence of DNA Adducts Combined with Epidemiological Approaches

The suspicion that ingestion of the* Aristolochia* species herbs may be responsible for the epidemic in Belgium was further corroborated by detection of aristolactam- (AL-) DNA adducts formed by metabolites of aristolochic acid (aristolactams) in samples of kidneys removed from five patients with nephropathy [36]. Cosyns et al. subsequently analyzed the urothelial lesions of kidneys and ureters removed during renal transplantation from 10 patients, overexpressed TP53 was observed, which suggested that a TP53 gene mutation plays a role for the carcinogenic effect [37]. Jelaković et al. extracted DNA from the renal cortex and urothelial tumor tissue of 67 patients that underwent nephroureterectomy for carcinomas of the upper urinary tract and resided in regions of Balkan endemic nephropathy. AL-DNA adducts and TP53 mutations were verified in tumor tissues of most patients. In contrast, neither AL-DNA adducts nor specific mutations were detected in tissues of patients residing in nonendemic regions [38]. Wu et al. investigated the chromosomal aberrations of UTUC specimens from seven dialysis patients in Taiwan by conventional comparative genomic hybridization and results showed that gains at 5p, 7, and 19q and losses at 4q, 9p, and 15q are common in UTUC of ESRD patients. In addition, female ESRD patients with UTUC had more frequent chromosomal aberrations than their male counterparts [39]. By whole-genome and exome analysis of nine AA-associated UTUCs in Singapore, Poon et al. found a high somatic mutation rate and the AA-induced mutations were also significantly enriched at splice sites, suggesting a role for splice-site mutations in UTUC pathogenesis [40]. Chen et al. analyzed 151 UTUC patients in a medical center of Taiwan and found that AL-DNA adducts were present in the renal cortex of 83% of patients with A : T to T : A mutations in TP53, FGFR3, or HRAS [41]. Results of the molecular epidemiology study were in coherence with the preceded finding in Belgium and therefore provided evidence for the biologic plausibility that the exposure to AA contributes to the development of UTUC.

### 2.5. Molecular Mechanisms of Renal Damage and Tumor Formation

AA-induced renal damage involves the tubular injury and the interstitium as well [34]. In acute phase, toxicity causes proximal tubule injury [42]. In chronic phase, the apoptosis of the tubular epithelial cells, defective activation of antioxidative enzymes, mitochondrial damage, impaired regeneration of proximal tubular epithelial cells, and interstitial cell proliferation lead to tubular atrophy and interstitial fibrosis [34, 43–45]. Because human urothelial tissue is rich in peroxidases, the aristolactams activated by peroxidase may result in the formation of the AL-DNA adducts in urothelial tissue [46, 47] and lead to A : T to T : A transverse mutations in the TP53 tumor suppression gene [38, 41, 47, 48], as demonstrated by overexpression of TP53 protein in patients with AAN and urothelial carcinoma [37]. Since the TP53 can promote cell-cycle checkpoints, DNA repair, and apoptosis [49], its mutation may play an important role for the AA-induced carcinogenic effect in human studies [37, 38, 41]. In rat model, AL-DNA adducts were also detected in the renal cortex of AA-treated rats [42]. In vitro studies, AA can induce AL-DNA adducts in proximal tubular cell line [50].

### 2.6. From Epidemiologic Observations to Causal Inference

**Figure 1:**
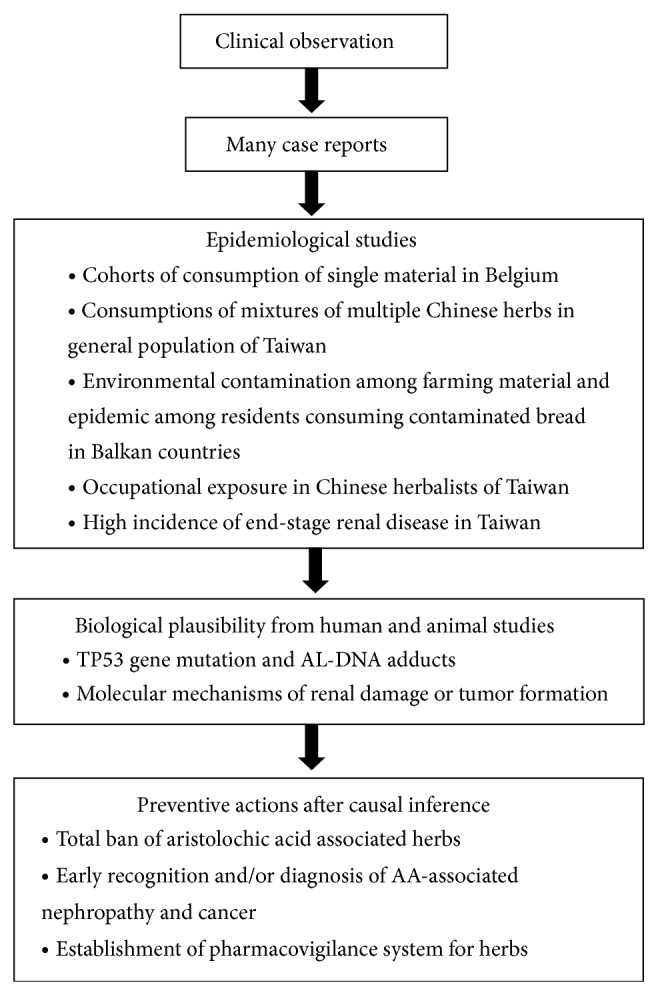
The sequence of establishing causality.

AA-induced kidney disease was once referred to as Chinese herbs nephropathy because of frequent occurrence in people taking Chinese herbal remedies, but it is now more accurately termed as aristolochic acid nephropathy (AAN) because the nephropathy is also observed in food contamination [51], occupational exposure [29, 30, 32], and so forth. Exposure to AA causes not only UTUC but also bladder cancer [52]. In determining whether an association is causal, the Hill criteria of causation are often applied [53, 54]. Clear temporal relationship between AA exposure and subsequently developed nephropathy and urological caner are observed in all epidemiological studies in Belgium and Taiwan. The association cannot be explained by alternative factors, including arsenic, cigarette smoking, and NSAID after appropriate control of confounding in these studies [22]. Moreover, a dose-response relationship was documented for AA exposure and UTUC based on the national-wide Chinese herbal pharmacoepidemiological studies in Taiwan, in which a threshold of herbal doses is also provided [22]. In different exposed populations in Belgium, Taiwan, and Balkan Peninsula, there are consistent and replicable findings that the AA exposures associate with increased risks for the occurrence of nephropathy and urothelial carcinoma. Moreover, a biologically plausible mechanism has been demonstrated by detection of gene mutations and DNA adducts, which appear to be specific in pathological finding and corroborates such a causal relationship (Figure 1). Thus, it is not a surprise that herbal remedies containing plant species of the genus Aristolochia have been designated to be a Group 1 carcinogen in humans [55].

## 3. Diagnosis of AA-Induced Nephropathy and Urothelial Carcinoma

### 3.1. Clinical Characteristics

AAN is characterized by anemia, mild tubular proteinuria, and initially normal blood pressure in approximately one-half of patients. Differences in disease progression may relate to the total dose of AA that was ingested [6]. Human subjects with high AA intake may progress to renal failure after 1 to 7 years, although subjects with a low cumulative AA intake can often maintain relatively normal kidney function over the course of 2–8 years of follow-up [56]. Based on a study including 39 patients with AAN who underwent prophylactic surgery, a cumulative dose of more than 200 g of Guang Fang Ji was associated with a higher risk of urothelial carcinoma [2]. Most of the cases of urothelial carcinoma were detected in AAN patients with ESRD. However, AA-induced urothelial carcinoma may occur without severe renal failure [57]. In contrast to the usual concept that urothelial carcinoma is more likely to develop in males than females [58, 59], the female gender has been shown to be associated with a higher risk of developing AA-related urothelial carcinoma [29, 60, 61].

### 3.2. Laboratory Findings

In AAN, microalbuminuria and the urinary excretion of beta 2-microglobulin appear to represent an early marker of tubular damage [62]. In such cases, there are also elevated levels of urine retinol-binding protein, urine N-acetyl-beta-glucosaminidase, anemia, and glucosuria [63].

### 3.3. Pathological Findings

Proximal renal tubular dysfunction and structural destruction would be the main positive findings in renal biopsy [63]. AAN has been classified as a separate entity of progressive tubulointerstitial nephropathy. The major pathological findings of AAN include hypocellular interstitial fibrosis, tubular atrophy, tubular brush border ablation, fibromyxoid or fibrous intimal thickening mainly of the interlobular arteries, mild to severe hyalinization, and sclerosis of the glomeruli decreasing from the outer to the inner cortex [63–67]. Urothelial malignancy is observed mainly in the upper urinary tract, such as the ureter and pelvis, during the first three years after exposure [37] but can also be observed in the bladder, with an approximately equal tendency, after longer follow-up periods [52]. The carcinogenesis of AA is associated with specific A : T to T : A mutations in the TP53 tumor-suppressor gene [37, 68–70].

### 3.4. AA Detection Method

To verify the exposure to AA, ultra-high-performance liquid chromatography-multistage fragmentation mass spectrometry can be applied to determine the presence of aristolochic acid I (AA I) and aristolochic acid II (AA II) in herbal dietary supplements [71]. Yang et al. reported a hollow fiber liquid-phase microextraction technique in conjunction with high-performance liquid chromatography for the extraction and quantitation of aristolochic acid I in human urine samples [72]. Following metabolic activation, AAs can form AL-DNA adducts in the renal cortex or ureteral tissues, which can serve as biomarkers of exposure to AA [2, 36, 38, 48, 73]. In addition, the ^32^P-postlabelling method can be used to detect AL-DNA adducts [74], although new liquid chromatography-mass spectrometry (LC-MS) methods have been applied in human renal cortex tissues [73, 75]. A noninvasive and efficient method using ultraperformance liquid chromatography-triple quadrupole mass spectrometry has also been developed to detect AL-DNA adducts in exfoliated urothelial cells [73, 76].

### 3.5. Treatment and Follow-Up

Steroid therapy was shown to slow the progression of renal failure in AAN [77, 78]. Regular medical follow-up is necessary for Chinese herbalists and those who have taken AA-containing herbal remedies. Because the prevalence of urothelial carcinoma among patients with end-stage AAN is high [2], prophylactic surgery to remove the ureter and kidney has been performed in patients with end-stage AAN in Belgium [37]. Because the exposure to AA increases the risk for bladder cancer [22, 27, 52], yearly screening by cystoscopy and computed tomography is recommended in patients with AAN [79, 80].

## 4. From Observational Studies to Preventive Actions

There has been an increase in the use of Chinese herbal remedies in Taiwan [14] and possibly worldwide [81]. Thus, AA epidemics have a significant implication for the safety of herbal remedies. Moreover, the misuse or substitution of Chinese herbs is common due to the inconsistent nomenclature system and the similarity of the names and appearance of many herbs [82]. For example, Han Fang Ji is often replaced by Guang Fang Ji, Mu Tong by Guan Mu Tong, and Mu Xiang by Qing Mu Xiang [82–85]. Although Chinese medicine is regarded as a formal medical treatment in the health care system of Taiwan [86] and Chinese herbal remedies are manufactured in certificated pharmaceutical factories, adulteration remains common due to the loose regulations and enforcement requiring factories to validate relatively few items. To proactively prevent the adverse reactions caused by Chinese herbal remedies and protect the public, Chinese herbal remedies and online herbal products must be regularly monitored by the Food and Drug Administration (FDA) to ensure that products are evaluated for their safety before marketing. Proper labeling and good surveillance systems will further protect consumers. Moreover, incorporating Chinese herbal drugs into the adverse drug reaction or poison surveillance system may be another effective way to achieve this goal [87]. In clinical practice, we recommend that physicians always keep in mind the possible consumption of herbs when treating a patient with renal disease or urological cancer. Although the US FDA bans all botanical remedies known or suspected to contain AA, AA I and AA II were detected in 20% and 7%, respectively, of tested samples in a survey of thirty herbal products marketed in the US via the Internet [71]. Xixin, which contains minute amount of AA [10, 88, 89], is still widely used in Asia, including Japan, Korea, China, and Taiwan, because Chinese medicine practitioners claim that its toxicity is negligible. However, AAN associated with Xixin was reported [15]. We suggest that the governments must adopt more restrictive regulations to eliminate all AA-containing herbs to protect public health.

In Chinese medicine, toxic herbs are seldom prescribed or used alone; in fact, traditional Chinese remedies are usually prescribed as complex mixtures of several different medicinal plants based on the ancient principle of “sovereign, minister, assistant, and courier,” which assigns each ingredient in a prescribed Chinese herbal formula a unique role so that the combination of them can enhance the main effect and reduce the toxicity of herbs [18, 19]. For example, minerals are commonly used in Chinese herbals remedies, and Chinese herbalists believe that the toxicity of minerals can be removed by traditional processing operations, including heating and quenching in vinegar, referred to as “pao zhi” [90]. However, no scientific evidence has shown that toxic contents, such as AA or heavy metals, can be eliminated by these methods. As the Chinese herbal products containing AA have been consistently shown to be associated with urothelial carcinoma, we must inform the general public, Chinese medicine practitioners, and healthcare professionals of proactively taking necessary precautions to avoid exposure to AA associated Chinese herbal products, establishing a pharmacovigilance system for traditional herbs, and continuing monitoring those who were exposed before. Because Taiwanese people may obtain herbs and herbal medicines from many different sources [3, 91], physicians shall still keep on watching potential nephrotoxicity of medicines even after AA-associated herbs were mostly removed from Chinese herbal products prescribed under the National Health Insurance system.

## Figures and Tables

**Table 1:** A list of Chinese herbs containing aristolochic acids.

Chinese herbal name	Botanical name	Chinese herbal formula containing this herb
Guang Fang Ji (Fangchi)	*Aristolochia fangchi *	Shu Jing Huo Xue Tang Shang Zhong Xia Tong Yong Tong Feng Fang Ji Huang Qi Tang Xiao Xu Ming Tang Jie Geng Tang Mu Fang Ji Tang

Xixin	*Radix et Rhizoma Asari *	Chuan Qiong Cha Diao San
		Xiao Qing Long Tang
		Du Huo Ji Sheng Tang

Guan Mu Tong	*Aristolochia manshuriensis *	Long Dan Xie Gan Tang
		Xin Yi San
		Ba Zheng San Gan Lou Xiao Du Dan Dao Chi San Dang Gui Si Ni Tang Mu Tong Guo Qi Yin Xiao Ji Yin Zi Ju He Wan Zheng Gu Zi Jin Dan

Qing Mu Xiang	*Aristolochia cucurbitifolia *	Xiang Sha Liu Jun Zi Tang
		Gui Pi Tang
		Zheng Gu Zi Jin Dan

Ma Dou Ling	*Aristolochia debilis *	

Tian Xian Teng	*Aristolochia contorta *	
